# Determinants of coping styles of people with Parkinson’s Disease

**DOI:** 10.1038/s41531-023-00548-3

**Published:** 2023-06-27

**Authors:** Eva M. Prins, Angelika D. Geerlings, Yoav Ben-Shlomo, Marjan J. Meinders, Bastiaan R. Bloem, Sirwan K. L. Darweesh

**Affiliations:** 1 Radboud University Medical Center; Donders Institute for Brain, Cognition and Behaviour; Department of Neurology, Center of Expertise for Parkinson & Movement Disorders, Nijmegen, The Netherlands; 2 Department of Population Health Sciences, Bristol Medical School, University of Bristol, Bristol, BS8 1NU UK; 3 Radboud University Medical Center; Radboud Institute for Health Sciences, Scientific Center for Quality of Healthcare, Nijmegen, The Netherlands

**Keywords:** Quality of life, Parkinson's disease

## Abstract

Little is known about how people with Parkinson’s disease (PD) cope with stressful life events. We examined the determinants of specific coping strategies and whether specific choices have any impact on quality of life (QoL). We recruited patients with PD who had been seen at a neurology outpatient clinic at least once during the past year as part of the PRIME-NL cohort study. Coping was measured using the Ways of Coping Questionnaire (WCQ) and QoL was measured using the Parkinson’s Disease Questionnaire (PDQ-39). 977 out of 988 participants completed the questionnaires and 935 participants were diagnosed with PD. Factor analysis was undertaken to test if ways of coping were similar or different to previous findings in a PD population. We used linear regression analyses to examine predictors of coping styles. We then used multivariable linear regression to test how coping style was associated with the domains of QoL conditional on potential confounders. The five coping styles identified by the factor analysis were: “taking action and emphasizing the positive”, “distancing and fantasizing”, “goal oriented and planful problem solving”, “seeking social support” and “avoidance and acceptance”. Age, gender, education and anxiety were associated with the type of coping strategy. For example, higher education was associated with more active coping strategies (e.g. β = 4.39, *p* < 0.001 for goal oriented). Conditional on other confounders, most coping strategies had little effect on QoL domains. These findings demonstrate that coping behavior of people with PD is influenced by psychological status and personal traits. However, there was only a modest effect of coping behavior on QoL. Future research needs to test whether the enhancement or discouragement of certain coping strategies is feasible and can enhance QoL.

## Introduction

Parkinson’s disease (PD) is a progressive and chronic neurodegenerative disease that is characterized by both motor symptoms—such as bradykinesia, tremor, and rigidity—and non-motor symptoms, including decreased cognitive functioning, fatigue, hallucinations, and depressive symptoms^1^. Previous research revealed that as the disease progresses, the impact of PD on daily lives increases, and consequently, people with PD experience the need to find new ways to adapt to new changes and challenges^2^.

Coping strategies can be considered as a person’s cognitive and behavioral efforts to maintain a state of normalcy when dealing with stressful events^3,4^. These can generally be classified as active or passive strategies^5^. Active coping, such as seeking support, is characterized by increased cognitive and behavioral attempts to actively confront the stressful situation and make environmental changes to solve the problem^6^, whereas passive coping, such as avoiding and denial, reflects inactivity and is associated with efforts to detach oneself from the problem^6–8^.

Previous studies have provided key clues on coping strategies used by people with PD. Specifically, active coping has generally shown to be more effective, the use of a certain coping strategy depends on many factors, including personality, life events, access to (health) resources, and disease severity^9–11^. However, it is unknown to what extent the choice of a coping strategy depends on how someone assesses the situation (s)he is confronted with and the available resources and the influence of the severity of their PD. We therefore aimed to assess the interplay of coping behavior of people with PD with different social demographics and disease-related characteristics.

The second aim was to investigate to what extent the choice of coping strategy affects the health-related quality of life (QoL) of a person with PD. Previous studies have found that people using active coping strategies generally have a better QoL^4,5,12^, whereas passive coping strategies are more often linked to worse depressive symptoms, more severe motor impairments and diminished QoL^12–14^. Therefore, we hypothesize that active coping strategies will be associated with greater QoL in people with PD. Knowing an individual’s choice of coping strategy makes it easier for health care professionals to tailor care to the unique needs of a person with PD.

## Results

### Patient characteristics

**Fig. 1:**
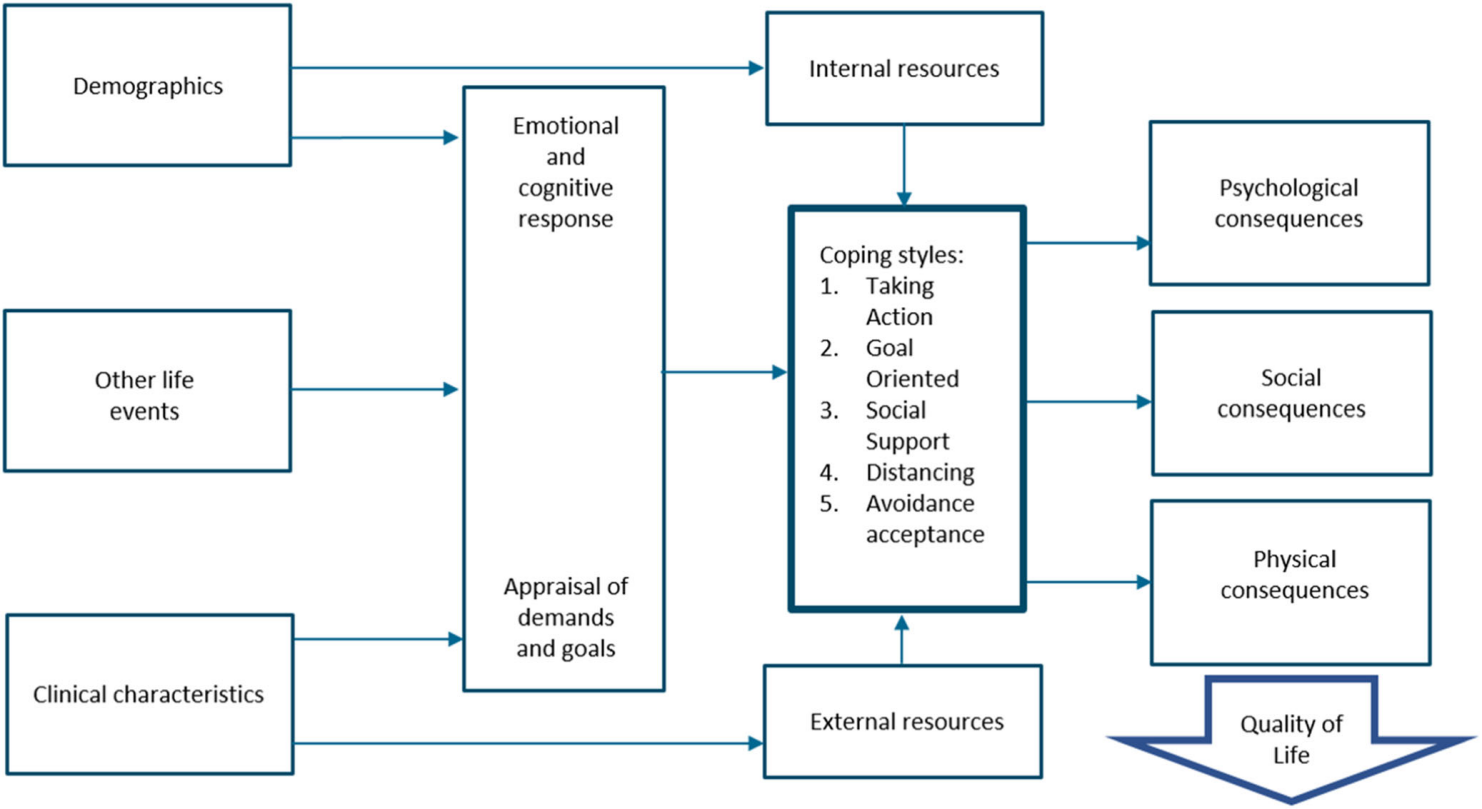
a schematic representation of the main results. An adaptation of the stress-coping model by Maes et al.^15^.

The total sample consisted of 988 patients. 11 people that did not complete all included questionnaires were excluded and 42 people were diagnosed with a form of atypical parkinsonism (AP) leaving data on 935 subjects. The sociodemographic and disease characteristics of the study sample are shown in Table 1. The mean age was 71.0 years (SD = 8.0), and 39% were women. Most people were married and lived together with their partner. Reasons for not having a job include retirement or losing a job due to PD, including being declared incapacitated. Figure 1 is a schematic representation of the main results^15^.

**Table 1:** Sample characteristics (*N* = 935).

Age, years, mean ± SD	71.0 ± 8.1
Female	368 (39)
*Education*^a^	
No formal education	4 (0.4)
Primary education	34 (4)
Secondary education	270 (29)
Higher	627 (67)
*Partnership*^b^	
Married	725 (78)
Living with partner	62 (7)
Divorced	35 (4)
Widowed	58 (6)
Single/Unmarried	50 (5)
Other	5 (0.5)
*Work status*	
Paid work	115 (12)
Unpaid work	48 (5)
No work	820 (88)
Retired	650 (79)
Declared incapacitated	151 (18)
*Living situation*^c^	
Alone	132 (14)
With partner	780 (83)
Other	23 (2)
Time since diagnosis, years, mean ± SD	7.3 ± 5.3

All values are *n* (%), unless stated otherwise.

^a^Missing in 1.2%.

^b^Missing in 1.1%.

^c^Missing in 0.4%.

### Stress situations

We examined the stress situations people were thinking of while answering the WCQ. The most common cause of stress was related to family: a total of 419 people (45%) experienced stress due to general family issues. Another very often mentioned stress resource was the person’s own or someone else’s health issues (43%). Furthermore, 119 people (13%) experienced stress due to the passing of someone close to them. Other, non-family-related causes of stress included work (10.1%), administrative issues such as money or housing (14%), or the COVID-19 pandemic (2.6%).

### Coping strategies

#### Factor analysis

The factor structure of the WCQ was examined to identify the coping strategies used within the Dutch PD sample. Both the original 8-factor and the 6-factor structure showed a poor fit (CFI = 0.706 and 0.649, respectively). Therefore we performed our own EFA which found 18 factors with an eigenvalue greater than one. The eigenvalue criteria indicated a suitability for a 4-factor structure. After rotation, model explained 27.5% of the variance. 23 items that that had a loading ≤0.35 and 3 items that loaded ≥0.35 on two factors were excluded. The reliability of the factors was tested using Cronbach’s alpha. All factors of the 4-factor structure showed Cronbach’s alpha ≥ 0.70, indicating that internal consistency was established.

However, when analyzing the reliability of the factor loadings, Cronbach’s alpha was below the minimum of 0.60 for all four factors, indicating that one of the scales might measure two distinct factors. Therefore, to improve the model, an EFA with a 5-factor structure was run, which showed a better fit to the data (see Table 2). The fifth factor had an alpha of 0.65 (SD = 0.03), not adhering to the criterium of Cronbach’s alpha ≥ 0.70, but the items showed better coherence between the items that loaded onto the different factors, favoring the 5-factor structure.

**Table 2:** Promax rotated loadings for a 5-factor exploratory analysis for the Ways of Coping Questionnaire (*N* = 935).

	Taking action and emphasizing the positive	Distancing and fantasizing	Goal oriented and planful problem solving	Seeking social support	Avoidance and acceptance
*Tried to do something about the problem in a creative way*	0.426				
*Changed as a person in a good way*	0.533				
*Realized I had caused the problem myself*	0.421				
*Felt better after this experience than before*	0.714				
*Gained new confidence*	0.633				
*Changed something for the better*	0.508				
*Stood my ground and fought for what I wanted to achieve*	0.405				
*I vowed that something like this would never happen to me again*	0.427				
*Changed something about myself*	0.518				
*In hindsight thought it was a good experience*	0.734				
*Hoped for a miracle*		0.432			
*Let it get to me*		0.655			
*Avoided others*		0.496			
*Closed myself off and tried not to think about it*		0.494			
*Kept others from knowing*		0.518			
*Wished situation would go away*		0.662			
*Daydreaming*		0.450			
*Wished the situation would disappear or be over in some way*		0.693			
*Had fantasies or wishes on how it could end*		0.548			
*Prepared for the worst*		0.446			
*I thought about what I would say or do*		0.357			
*Focus on the next step*			0.717		
*Tried to analyze the problem to understand it better*			0.717		
*Thought of several solutions for the problem*			0.355		
*Tried to calmly consider my options and not follow my first instinct*			0.521		
*Talked to someone about the situation*				0.496	
*Accepting sympathy/understanding*				0.599	
*Talked to someone who could do something about the problem*				0.439	
*Asked for advice from someone I respect*				0.589	
*Talked to someone about my feelings*				0.662	
*Thinking situation would go away by itself*					0.564
*Tried to get something positive out of the situation*					0.365
*Tried not to commit to anything*					0.443
*Resigned myself to my fate*					0.499
*Acted as if nothing had happened*					0.472
*Accepted the situation because nothing could be done*					0.476

On the left the different statements of the WCQ are shown. The numbers represent the loadings of these statements on the five different factors. The tables includes loadings only if they were ≥0.35, and loaded on only one factor. *p* ≤ 0.05.

The five factors were labeled on their content; factor 1 (12 items) was labeled “taking action and emphasizing the positive” (Cronbach’s α = 0.81); factor 2 (10 items) was labeled “distancing and fantasizing” (Cronbach’s α = 0.79); factor 3 (6 items) was called “goal oriented and planful problem solving” (Cronbach’s α = 0.74); factor 4 (6 items) was named “seeking social support” (Cronbach’s α = 0.77); factor 5 (7 items) was called “avoidance and acceptance” (Cronbach’s α = 0.65). An overview of the five coping styles and their description can be found in Supplementary Table 1^16^.

#### Intercorrelation of the coping dimensions

The factors showed low to moderate correlation (see Table 3). Low correlations were found for avoidance-acceptance with distancing and fantasizing (*r* = 0.09) and seeking social support (*r* = 0.09). Relatively high correlations were discovered for goal oriented and planful problem-solving with taking action and emphasizing the positive (*r* = 0.58), and distancing and fantasizing (*r* = 0.31).

**Table 3:** Correlation levels of the five coping strategies.

	Avoidance	Distancing	Social Support	Taking Action	Goal Oriented
Avoidance	1.00	0.0875	0.0876	0.209	0.237
Distancing		1.00	0.273	0.231	0.306
Social support			1.00	0.408	0.407
Taking action				1.00	0.584
Goal oriented					1.00

#### Determinants of coping strategies

We found that age, gender, educational level, anxiety and depression were all associated with coping strategies (see Supplementary Table 2). Work status, partnership, disease duration and severity of motor symptoms were not significantly associated with the five ways of coping. We explored these associations further using multivariable linear regression (see Table 4). “Taking action” was associated with secondary education but the full model explained only 1.6% of the variance. Women, anxiety and depression were associated with “distancing” explaining 19.5% of the variance for this strategy. Younger people with a higher level of education compared to primary education show more “goal oriented behavior” (6.5% of the variance). Older adults were less likely to seek social support whilst a higher level of anxiety was positively associated (4.7% of the variance) hence younger people with higher levels of anxiety use this strategy more often. Lower levels of anxiety were associated with “avoidance acceptance” (2.4% of the variance), as well as age.

**Table 4:** Levels of association of the determinants with the coping strategies.

	Age		Gender^a^		Secondary education		Higher education		Anxiety		Depression	
	β	p	β	p	β	p	β	p	β	p	β	p
**Taking action**	–	–	–	–	−0.14 (−0.21;−0.06)	0.00015	–	–	–	–	–	–
**Distancing**	–	–	0.10 (0.03;0.16)	0.0041	–	–	–	–	0.01 (0.004;0.01)	<0.001	0.02 (0.01; 0.03)	<0.001
**Goal-oriented**	–	–	–	–	–	–	0.44 (0.24;0.6)	<0.001	–	–	0.006 (0.00;0.01)	0.024
**Social support**	−0.01^b^ (−0.02;−0.004)	<0.001	0.21 (0.11; 0.30)	<0.001	−0.20 (−0.30; −0.09)	0.00020	–	–	–	–	–	–
**Avoidance**	0.005 (0.001;0.008)	<0.001	–	–	–	–	–	–	−0.003 (−0.004; −0.002)	<0.001	–	–

Negative or positive association between the different determinants and the coping strategies.

If β is positive, the interpretation is that for every 1-unit increase in the predictor variable, the outcome variable will increase by the β value. Only significant values are presented in the figure. p-value is set at 0.05.

Age is in years; anxiety is measured using the State Trait Anxiety Inventory; depression is measured using the Beck Depression Inventory. The maximum values for age, anxiety and depression in our study population were: 94 years (for age), 44 points on the Beck Depression Inventory (for depression), and 144 points on the State Trait Anxiety Inventory (for anxiety).

^a^1 = male; 0 = female.

^b^This is the only non-linear relationship in this table.

#### Relation between quality of life and coping

Most of the differences in QoL were predicted by patient and disease characteristics, with weaker associations seen with coping strategies (see Table 5). In the full models, we found that “taking action was not predictive of any of the QoL domains; “distancing” was associated with worse emotional well-being and stigma; Goal orientation was associated with worse bodily discomfort; Social support was associated with worse emotional well-being, better communication and worse bodily discomfort; Avoidance acceptance was associated with worse cognitions. Women did worse than men on the dimensions of “communication” and “cognition”, but had higher QoL levels on “mobility”, “emotional well-being” (EWB), “stigma” and “bodily discomfort” (BD). Older people had a lower QoL for mobility than younger people, while younger people experienced more of a negative effect on the levels of stigma and BD. A higher level of education was associated with a higher QoL for mobility and ADL. Having a partner resulted in lower levels for communication and cognitions. Anxiety and depression levels as well as levels of motor symptoms had negative effects on almost every dimension of QoL. Work status did not have any effect on the dimensions. An interaction test between work status and QoL amongst subjects <65 and >=65 years did not result in major group differences. The results of this interaction test are presented in Supplementary Table 3.

**Table 5:** Associations between different patient characteristics and coping strategies with the dimensions of QoL.

	Mobility			ADL			EWB			Stigma			Cognitions			Communication			BD		
	I	II	III	I	II	III	I	II	III	I	II	III	I	II	III	I	II	III	I	II	III
Age	8.30***	3.54***	3.71***	6.91***	0.48	0.48	0.70	−1.58	−0.90	1.78	−1.31	−1.26	2.69**	0.88	0.76	3.76***	−0.54	−0.55	−0.35	−3.21**	−3.03**
Gender	7.75***	10.55***	9.99***	0.27	1.47	1.20	6.46***	6.13***	5.27***	2.17*	1.48	1.22	−2.71**	−3.69***	−3.79***	−3.01**	−3.34***	−3.09**	6.64***	6.43***	5.86***
Secondary education		−2.33*	−2.14*		−2.51*	−2.49*		−1.16	−0.99		−1.45	−1.43		−1.96	−2.03*		0.00	−0.10		−0.29	−0.31
Higher education		−3.28**	−3.23**		−3.41***	−3.34***		−1.31	−1.44		−1.52	−1.56		−1.53	−1.69		0.44	0.50		−0.94	−1.27
Partnership		0.18	0.29		0.23	0.29		−0.29	0.03		0.00	0.06		2.98**	3.03**		2.91**	2.83**		−0.83	−0.69
Work status		−0.69	−0.68		0.58	0.79		−0.28	−0.20		1.48	1.43		−0.06	−0.21		−1.05	−1.38		−1.94	−1.86
Time since diagnosis		3.18**	3.16**		1.79	1.96		−1.57	−1.47		0.22	0.29		0.25	0.39		2.86**	2.58**		−0.18	0.08
Anxiety		4.30***	4.12***		2.55*	2.31*		13.74***	13.03***		7.69***	7.28***		3.64***	3.81***		2.36*	2.17*		2.43*	2.39*
Depression		2.77**	2.71**		−3.16**	−3.10**		10.52***	10.00***		2.42*	1.76***		8.01***	7.93***		4.04***	3.79***		4.00***	3.55***
Living situation		−0.62	−0.71		−0.40	−0.50		0.02	−0.20		−0.12	−0.20		−2.56*	−2.62**		−2.30*	−2.18*		1.25	1.11
Motor symptoms		22.27***	21.90***		32.79***	32.55***		4.48***	4.11***		7.32***	7.08*		5.35***	4.99***		13.95***	13.69**		6.20***	6.24***
Taking Action			0.71			−1.11			−0.32			1.03			0.97			1.58			−0.88
Distancing			−0.15			0.19			2.29*			2.68			−0.85			1.44			0.28
Goal Oriented			−0.54			0.29			0.28			−0.71			0.31			−0.98			1.72
Social Support			1.67			1.09			4.21***			0.14			0.33			−2.07*			1.81
Avoidance Acceptance			0.61			−0.92			0.76			1.84			2.45*			1.51			0.63

I: control model, only including age and gender; II: including all other demographics; III: the full model, including all demographics and coping strategies; IV: including the coping strategies, but without anxiety and depression.

Age is in years; anxiety is measured using the State Trait Anxiety Inventory; depression is measured using the Beck Depression Inventory. The maximum values for age, anxiety and depression in our study population were: 94 years (for age), 44 points on the Beck Depression Inventory (for depression), and 144 points on the State Trait Anxiety Inventory (for anxiety).

*ADL* activities of daily living, *EWB* emotional well−being, *BD* bodily discomfort.

*1 = male; 0 = female.

***p ≤ 0.001, **p ≤ 0.01, *p ≤ 0.5.

In our sensitivity analysis we found that excluding anxiety and depression from our models revealed new associations so that distancing was now negatively associated with mobility, cognitions, communication and BD whilst avoidance acceptance was associated with better emotional well-being.

We also considered whether the lack of associations of some variables (e.g., work status or partnership) could be explained by the fact that models I-III were all adjusted for gender. Therefore, we repeated the analysis without adjustment for gender (see Supplementary Table 4). We observed no meaningful changes in the results.

#### The impact of COVID-19

As the data were collected during the COVID-19 pandemic, an additional questionnaire about the impact of COVID-19 on mental health and access to healthcare by people with PD was added at a later stage. In total, 822 have completed this additional questionnaire (83% of the total sample). 274 (33%) people who completed the questionnaire experienced a large impact of the COVID-19 pandemic on their access to care, while 600 (73%) people indicated that they experienced a large impact on their social life. 204 (25%) people experienced a low COVID-19 burden. We repeated the analyses stratifying by whether the subjects reported experiencing a high or low impact of COVID-19 but did not find any group differences on QoL so have not presented these results.

#### The five assumptions for a linear model

We checked the five key assumptions for a linear model. We verified that the associations had a linear relationship (with one exception—see below), multivariate normality, no or little multicollinearity, no auto-correlation and homoscedasticity. We assessed non-linear associations by separately adding a quadratic term for each determinant to the model. We considered an association of a quadratic term with a coping strategy with *p* < 0.05 to be suggestive of a non-linear association. Across all analyses, one determinant had a non-linear association with a single coping strategy: we observed an association between a quadratic term of age and ‘seeking social support’.

## Discussion

In the present study, we identified several determinants of coping strategies for persons with PD. We also investigated the relationship between these coping strategies and QoL. For this purpose, we re-examined the proposed factor structure of the WCQ and found that in our population, a simpler five-factor was more appropriate. We also found that age, gender, educational level, anxiety, and depression were all associated with different coping strategies. However, compared to demographic and disease-related factors, only “distancing”, “goal orientation” and “social support” were associated with worse QoL domains, albeit more modestly. More associations were found if we did not adjust for anxiety and depression as some strategies are partially a result of these traits.

Against our expectations, no effects on coping were found for partnership and work status. We expected that people with a partner and/or a job would use more effective coping strategies, as these people tend to have to make less changes in lifestyle and have a lower impact of living with a chronic illness^12,17^. Our sample included fairly old participants, as is to be expected in a PD population. The lack of associations of work status and partnership with coping strategies may be explained by the relatively limited number of individuals who were still working or who did not have a partner in our study. Work status might have a bigger impact in a younger population, as older people are usually retired. A previous study^18^ researched the effect of chronic pain on coping. They found that a better work status might be a consequence of better coping with chronic pain rather than a cause for better coping, as better coping is associated with a better work status. However, an interaction test between work status and QoL among subjects <65 and > = 65 years did not reveal big differences between the two subgroups. The small effect of work status and partnership on coping strategies can also be linked to the small sample size of these groups. A large part of our population does not work anymore and lives with their partner.

People without a partner might use the coping strategy of seeking social support to compensate for the absence of a partner. A stable partnership is associated with better physical and mental health outcomes possibly due to better emotional and social support if partners listen and provide empathy^19,20^. A previous study^12^ failed to find differences in coping styles between single and married participants. In both studies, there were few subjects without a partner, so these may have been underpowered. Another possible explanation is that children or care givers serve as proxy partners for single participants. For future studies, it might be interesting to also explore the coping styles of carers of people with PD, as it has been shown that the carer burden has a large impact on coping and QoL^21^.

We found that gender was an influential demographic variable. A previous study pointed out that men seem to use more practical, active ways of coping as they tend to suppress their emotions due to societal views of masculinity^4,22^, while women seek social support whilst using other coping strategies. Even though women tend to use more emotion-focused strategies, they tend to combine these with effective active strategies. Therefore, the mix of active and passive coping strategies associated with gender is not surprising.

We expected that there would be age differences in active and passive coping. The results revealed that it is more likely that younger people seek social support and show more goal-oriented behavior, whereas elderly more often use avoidance and acceptance. The more passive coping in older people might be due to the fact that they have lived with the disease for a longer time, have multiple co-morbidities and frailty. This is contrary to previous findings^23^, as these pointed out that younger people with PD more often show less mature coping compared to elderly, and maturity is often associated with more problem-focused coping. A higher level of education, such as university or a PhD, is significantly associated with goal-oriented behavior, as might be expected. Active coping behavior is associated with higher levels of cognitive performance and therefore with higher levels of education, contrary to passive coping^12^. However, the present study only included middle-aged people and elderly. Therefore, the impact of age differences on ways of coping needs to be further investigated in future research.

Previous literature has already shown how coping behavior can have psychological, social and physical consequences^4,5,12–14^. To examine this, we studied the interaction between coping styles and QoL. We expected, a priori, to find a different association between active and passive coping behavior on QoL. Most coping strategies had a minimal association with QoL. Distancing and seeking social support had the largest influence on QoL. Seeking social support is an active coping strategy and is associated with greater overall well-being^24^, while a passive strategy such as distancing is often associated with lower QoL^3,5^. It has been shown that persons with PD often use multiple coping strategies in a certain situation^12^. Deployment of coping strategies will differ across situations, implying it might be better to consider a global composite score^25^ rather than the individual components as predictors of QoL.

Levels of anxiety and depression have negative impacts on all dimensions of health-related QoL. A previous study pointed out the importance of taking these two variables into account besides physical symptoms, as they have such a big, mostly negative, influence on QoL^26^. It is quite plausible that one’s coping style is partially determined by anxiety and depression which would result in reduced social interaction. Our models without these predictors did indeed find more associations with distancing and avoidance acceptance. The strategy of avoidance and acceptance was associated with lower levels of anxiety in our study, which is somewhat counterintuitive. A possible explanation is that individuals who tend to accept impairments in their daily activities due to PD relatively easily might also be less anxious about the prospect of acquiring additional impairments in the future. Another possible explanation could be that lower levels are a result of the avoidance strategy, instead of as a cause (reverse causation).

A main strength of our study is the relatively large sample size of people with PD as well as the high proportion of women participating in this study. Previous research has revealed that women are underrepresented in Parkinson’s studies^12,27^. Additionally, although many of the demographics included in this study had already been studied previously, little research includes all of them in one study. In this way, coping behavior and its association with QoL can be meaningfully explained by many different factors.

There are several limitations that need to be considered. Our results are based on cross-sectional data, so we cannot determine the temporal relationship between variables. Some associations might reflect reverse causation, so for example, emotional well-being may determine “distancing” rather than vice versa. This survey overlapped the current COVID-19 pandemic and this may have impacted our results. However, a separate analysis including a questionnaire on the impact of COVID-19 did not meaningfully change our results. A previous PRIME-NL study found that women, people with a higher educational level and people using distancing or seeking social support as a coping strategy experienced a higher impact of the COVID-19 pandemic^28^. Our study was fairly homogeneous, which could make our findings less generalizable to other PD populations. The WCQ does not distinguish between PD and non-PD-related stressful situations^3^. We would get a better insight into the way people with PD use coping strategies if people were instructed to think of how they respond to PD-specific or non-PD-specific stressful situations. Similarly, it has been suggested that one should adjust for the type of stressful event (which may or may not be under one’s control), when examining the association between coping strategy and QoL^14^. Another limitation is that we lacked detailed information on key disease severity indicators, such as cognitive impairment, self-efficacy^29^, severity of non-motor symptoms^30,31^ or measures of social support and quality of care that might influence coping behavior. Also, given that this was a survey-based study, we had limited data to classify clinical subtypes of PD. We cannot exclude that these limitations may have influenced the results.

In summary, we have found fewer coping strategies in our PD population and that a general model of coping is hard to create as it is dependent on many different facets^32^. Overall QoL is more strongly determined by clinical and demographic factors than coping styles, though the former may be less amenable to intervention. Future research needs to test whether the enhancement or discouragement of certain coping strategies is feasible and can enhance QoL. Currently, no firm recommendations can be made to health care professionals as to how they can incorporate an individual’s coping strategy within the framework of personalized health care provision.

## Methods

### Participants

This cross-sectional study was embedded within the PRIME-NL Parkinson Evaluation study, a prospective observational study among people with parkinsonism, informal caregivers and healthcare professionals in the Netherlands^33^. The study has been approved by the ethics committee of the Radboud university medical center (file number 2019–5618). In order to be eligible, participants had to have (i) a clinical diagnosis of PD; and (ii) visited the neurology outpatient clinic of a community hospital at least once during the past year. All participants gave written informed consent. Various differential recruitment strategies were used^33^, so the exact response rate is unclear. The assessment took place between January 2020 and February 2021. The data were collected through self-completed online questionnaires.

### Measures

#### Outcome and key exposure measures

##### The ways of coping questionnaire (WCQ)

To measure coping, various coping scales have been developed over time^34^. This study will focus on the Ways of Coping Questionnaire (WCQ)^6^, an originally eight factor-structured questionnaire that has been widely used in both clinical and non-clinical groups, including other chronic diseases than PD such as stroke or multiple sclerosis^3^. It has been argued that the general WCQ might not be suited for every chronic disease but might be illness-specific instead^13^. In line with this hypothesis, a previous study found a six-factor structure of the WCQ for an Australian PD population^3^.

The WCQ consists of 67 statements regarding coping in a stressful situation^6^. The participant received the instruction to think of a stressful situation that occurred within the last 12 months, which did not have to be related to PD. The participants then had to indicate how much they thought each statement applied to their situation on a 4-point Likert scale ranging from 0 (“does not apply and/or not used”) to 3 (“used a great deal”). The most recent version of the WCQ consists of eight dimensions of coping: confrontive coping, distancing, self-controlling, seeking social support, accepting responsibility, escape-avoidance, planful problem-solving and positive reappraisal^35^. For our study, a validated translation of the Dutch WCQ was used^36^.

##### Parkinson’s disease questionnaire (PDQ-39)

QoL was measured using the Dutch version of the PDQ-39^37^. The PDQ-39 is a questionnaire used to assess quality of life for people with PD^38^. It consists of 39 items divided into eight dimensions: mobility, activities of daily living (ADL), emotional well-being (EWB), stigma, cognitions, communication, bodily discomfort (BD) and social support^39^. The items have a five-point scale, ranging from 0 (“never to always/cannot do it at all”) to 4 (“always”)^40^. The participants were asked to fill out the PDQ-39, and accordingly a general score for each dimension of QoL was calculated for each individual. The eight-dimension score is created by Likert’s method for summated rating. Each dimension score ranges from 0 to 100 with higher scores indicating worse QoL (0 = good health; 100 = poor health).

#### Sociodemographic variables

The following socio-demographic variables were included: gender, age, education level, work status and partnership. Work status was recoded to a binary variable, having a paid job Yes/No. Partnership was dichotomized into having a partner Yes/No. Education level was subdivided into three levels: primary education, secondary education, and higher education.

#### Disease-related variables

##### Non-motor symptoms: anxiety and depression

The State-Trait Anxiety Inventory for adults (STAI) was used to evaluate level of anxiety^41^. The STAI measures two dimensions: (1) state anxiety, which assesses the current emotional state of anxiety, and (2) trait anxiety, which refers to the type of anxiety characteristics for the individual’s personality. It is a self-reported questionnaire composed of 40 items and is based on a 4-point Likert scale ranging from 0 (almost never) to 3 (almost always). The total score of each dimension ranges from 20 to 80, and higher scores indicate greater anxiety. For the purpose of our study, the state anxiety was used only.

The Beck Depressive Inventory (BDI)^42,43^ was used to assess the level of depression per participant. The BDI is a 21-item self-reported questionnaire used to measure the severity of depressive symptoms. Each item is composed of four statements, each depicting a particular symptom. Participants can score on each item on a 4-point Likert scale ranging from 0 (no symptoms) to 3 (very intense symptoms). The total scores indicate minimal, mild, moderate, or severe depression.

##### Motor symptoms

Part II of the MDS-Unified Parkinson’s Disease Rating Scale (MDS-UDPRS) of the self-assessment patient questionnaire was used to assess motor aspect of experiences of daily living^44^. It is a 14-item self-reported questionnaire on which participants can score on a 5-point Likert scale ranging from 0 (“normal”) to 4 (“severe”).

#### Additional measure regarding the impact of COVID-19

As the baseline assessments of this study took place during the COVID-19 pandemic in the Netherlands, a standardized questionnaire about the experienced impact of COVID-19 on the lives of people with PD was included^33^. The questionnaire consisted of 8 items measuring COVID-19-related stress and was scored on a 6-point Likert-scale ranging from 0 (“this situation did not occur”) to 5 (“very troublesome”). Out of the full sample of 988 participants, 822 completed the questionnaire, and these people were included in a separate analysis to analyze the influence of the pandemic on the mental health of the participants and their access to health care.

### Statistical analysis

The original eight-factor structure of the WCQ and the six-factor structure previously found for a PD sample^3^ were tested using two confirmatory factor analyses (CFA). If the sample showed a poor fit (CFI < 0.95), exploratory factor analyses (EFA) and principal component analyses (PCA) were performed to find out which number of factors resulted in the best fit. Items that loaded highly on two factors and items that had a loading ≤ 0.35 were excluded. The fit of the models was evaluated using several criteria: the Comparative Fit Index (CFI) and Cronbach’s alpha, to test how closely related the items of the factor were. An alpha value ≥ 0.70 was considered desirable. An oblique rotation of the factor analysis was chosen to allow for the usage of more than one coping strategy, as it is expected that individuals do not only use one coping strategy, but rather score higher for one without the exclusion of others^45^. Correlation between the different coping strategies was checked by using Pearson’s *r* to examine the correlation between the strategies. This was done to account for the instability that the WCQ can cause as the results can differ per individual and per situation^9^.

To test our first hypothesis, multivariate analyses of variance (MANOVA) were performed to examine the effect of sociodemographic and disease related variables on coping strategies, using the coping strategies derived from the factor analysis as dependent variables. An overall relative score was calculated for each coping strategy per participant. Age, gender, education level, partnership and work status were included as sociodemographic variables and disease duration, level of anxiety and depressive symptoms, and motor symptoms as disease-related variables. We applied Pillai’s trace test as it has been shown to be the most robust to any violations of model assumptions for multivariate analysis^46^. We used multivariable linear regression models to analyze how the individual independent variables differed for the five coping strategies.

We used multivariable linear regression models to determine how the demographics and disease-related characteristics were associated with the 6 domains of the PDQ-39. We ran three sets of incremental models; (i) adjusting for gender and age, (ii) adding demographic and disease-related variables (iii) adding the coping strategies. As a sensitivity analysis, we repeated model (iii) but excluding the anxiety and depression covariates to see how much of the effect of the coping strategy was attenuated by these psychological variables. All statistical analyses were performed with R Studio version 1.1.463.

### Reporting summary

Further information on research design is available in the Nature Research Reporting Summary linked to this article.

## Supplementary information

Supplementary Material

Reporting Summary

## Data Availability

The data generated during the present study are available from the corresponding author for researchers who have a specific question that can be answered with this data.

## Code availability

The code generated for the analysis will be available upon request for researchers who have a specific question that can be answered with this data.
