# Supplementary material for: Determinants of coping styles of people with Parkinson’s Disease
Source: NPJ Parkinsons Dis. 2023 Jun 27;9:99. doi: 10.1038/s41531-023-00548-3 (PMC10300125; doi:10.1038/s41531-023-00548-3)
Supplement: Supplementary file 1 — Supplementary Material [file 41531_2023_548_MOESM1_ESM.pdf]

Supplementary table 1

Name	Description	Example of a statement
Taking action and emphasizing the positive	Looking for the 'silver lining' of things.	"I stand my ground and fight for what I want to achieve."
Distancing and fantasizing	Hoping that the problem will solve itself.	"I hoped for a miracle."
Goal oriented and planful problem solving	Making a plan of action and following it.	"I try to analyze the problem to understand it better."
Seeking social support	Accepting sympathy and understanding from someone.	"I talked to someone about my feelings."
Avoidance and acceptance	Accepting the situation as it is and not thinking too much about it.	"I accept the situation as nothing can be done."

Descriptions based on Folkman et al. (1980) [30]

Supplementary table 2

	Age		Gender		Secondary education *vs. primary education*		Higher education *vs. primary education*		Partnership		Work status		Time since diagnosis		Anxiety		Depression		Motor symptoms	
	F	p	F	p	F	p	F	p	F	p	F	p	F	p	F	p	F	p	F	p
Multivariate test (Pillai's trace)	6	<.01	9	<.01	9	<.01	5	<0.01	1	0.60	2	0.11	1	0.68	12	<.01	37	<.01	2	0.12
Univariate tests																				
Taking action	1.55	0.213	2.36	0.12	12.80	<.01	3.13	0.08	2.44	0.12	0.66	0.42	0.60	0.44	0.20	0.66	1.36	0.24	0.17	0.68
Distancing	1.93	0.17	16.6	<0.01	0.49	0.48	<0.01	0.94	0.43	0.51	1.55	0.21	0.67	0.41	30.80	<0.01	172.55	<0.01	0.24	0.63
Goal-oriented	7.36	0.007	<0.01	0.98	31.18	<0.01	19.40	<0.01	0.38	0.54	0.47	0.49	2.09	0.15	<0.01	1.00	5.83	0.02	0.99	0.32
Social support	16.73	<0.01	14.25	<0.01	13.98	<0.01	0.37	0.54	1.11	0.29	0.68	0.41	0.33	0.57	2.02	0.16	1.92	0.17	3.27	0.07
Avoidance	5.65	0.02	1.88	0.17	2.84	0.09	3.00	0.08	0.59	0.44	3.70	0.05	0.60	0.44	19.57	<0.01	1.24	0.27	0.65	0.42

MANOVA: Multivariate analysis of variance. This figure shows if there exist associations between the different determinants and the coping strategies. A sufficiently large F value indicates that the term is significant, meaning that there is an association. p-value is set at 0.05

Supplementary table 3

	Mobility		ADL		EWB		Stigma		Cognitions		Communication		BD	
	>=65	<65	>=65	<65	>=65	<65	>=65	<65	>=65	<65	>=65	<65	>=65	<65
Gender	9.61***	9.61***	1.16	1.16	5.38***	5.38***	1.34	1.34	-3.89***	-3.89***	-3.05**	-3.05**	6.15***	6.15***
Secondary education	-2.56*	-2.56*	-2.56*	-2.56*	-0.89	-0.89	-1.30	-1.30	-2.13*	-2.13*	-0.04	-0.04	0.04	0.04
Higher education	-3.74***	-3.74***	-3.44***	-3.44***	-1.33	-1.33	-1.40	-1.40	-1.81	-1.81	0.58	0.58	-0.86	-0.86
Partnership	0.27	0.27	0.28	0.28	0.03	0.03	0.07	0.07	3.03**	3.03**	2.84**	2.84**	-0.67	-0.67
Work status	-2.00*	-2.00*	0.67	0.67	0.10	0.10	1.95	1.95	-0.48	-0.48	-1.27	-1.27	-0.91	-0.91
Time since diagnosis	2.87**	2.87**	1.93	1.93	-1.41	-1.41	0.39	0.39	0.34	0.34	2.63**	2.63**	0.31	0.31
Anxiety	4.45***	4.45***	2.36*	2.36*	13.01***	13.01***	7.20***	7.20***	3.90***	3.90***	2.13*	2.13*	2.10*	2.10*
Depression	2.26*	2.26*	-3.18**	-3.18**	10.18***	10.18***	1.93	1.93	7.90***	7.90***	3.89***	3.89***	3.93***	3.93***
Living situation	-0.67	-0.67	-0.49	-0.49	-0.29	-0.29	-0.21	-0.21	-2.62**	-2.62**	-2.19*	-2.19*	1.08	1.08
Motor symptoms	22.94***	22.94***	33.33***	33.33***	4.02***	4.02***	6.97***	6.97***	5.25***	5.25***	13.87***	13.87***	5.72***	5.72***
Taking Action	0.74	0.74	-1.10	-1.10	-0.33	-0.33	1.02	1.02	0.98	0.98	1.57	1.57	-0.90	-0.90
Distancing	-0.34	-0.34	0.16	0.16	2.35*	2.35*	2.75**	2.75**	-0.89	-0.89	1.47	1.47	0.44	0.44
Goal Oriented	-0.58	-0.58	0.28	0.28	0.30	0.30	-0.70	-0.70	0.30	0.30	-0.97	-0.97	1.75	1.75
Social Support	1.34	1.34	1.05	1.05	4.30***	4.30***	0.25	0.25	0.27	0.27	-2.04*	-2.04*	2.07*	2.07*
Avoidance Acceptance	1.03	1.03	-0.87	-0.87	0.66	0.66	1.70	1.70	2.56*	2.56*	1.46	1.46	0.29	0.29

Supplementary table 4

	Mobility			ADL			EWB			Stigma			Cognitions			Communication			BD		
	III	IV	V	III	IV	V	III	IV	V	III	IV	V	III	IV	V	III	IV	V	III	IV	V
Age	3.71***	0.07	2.82**	0.48	0.74	0.86	-0.90	0.06	-1.35	-1.26	0.00**	-3.09**	0.76	0.37	-0.24	-0.55	0.40	-1.52	-3.03**	0.00***	-3.08**
Gender	9.99***		9.74***	1.20		0.95	5.27***		5.60***	1.22		2.74**	-3.79***		-2.87**	-3.09**		-2.97**	5.86***		6.43***
Secondary education	-2.14*	0.02*	-2.40*	-2.49*	0.01**	-2.53*	-0.99	0.29	-0.52	-1.43	0.17	-1.13	-2.03*	0.06	-1.62	-0.10	0.86	-0.08	-0.31	0.51	0.60
Higher education	-3.23**	0.00***	-3.80***	-3.34***	0.00***	-3.38***	-1.44	0.08	-1.74	-1.56	0.54	-0.82	-1.69	0.16	-1.85	0.50	0.54	0.27	-1.27	0.43	-0.78
Partnership	0.29	0.58	-0.20	0.29	0.72	-0.15	0.03	0.41	-0.60	0.06	0.68	-0.40	3.03**	0.00***	2.79**	2.83**	0.02*	2.12*	-0.69	0.14	-1.34
Work status	-0.68	0.15	-0.87	0.79	0.53	0.88	-0.20	0.66	0.18	1.43	0.97	0.28	-0.21	0.91	-0.30	-1.38	0.08	-1.91	-1.86	0.04*	-1.72
Time since diagnosis	3.16**	0.00***	2.01*	1.96	0.07	1.71	-1.47	0.60	-2.60**	0.29	0.01*	1.09	0.39	1.00	-0.71	2.58**	0.12	1.32	0.08	0.25	-0.06
Anxiety	4.12***	<0.001***		2.31*	0.01**		13.03***	<0.001***		7.28***	<0.001***		3.81***	0.00***		2.17*	0.05*		2.39*	0.01*	
Depression	2.71**	0.04*		-3.10**	0.00**		10.00***	<0.001***		1.76***	<0.001***		7.93***	<0.001***		3.79***	0.00***		3.55***	<0.001***	
Living situation	-0.71	0.86	-0.35	-0.50	0.97	-0.17	-0.20	0.69	-0.08	-0.20	0.55	-0.75	-2.62**	0.00**	-2.65**	-2.18*	0.19	-1.33	1.11	0.08	1.69
Motor symptoms	21.90***	<0.001***	27.94***	32.55***	<0.001***	36.87***	4.11***	0.00***	11.40***	7.08*	0.06	6.74***	4.99***	<0.001***	10.03***	13.69**	<0.001***	18.85***	6.24***	<0.001***	9.06***
Taking Action	0.71	0.37	0.01	-1.11	0.22	-1.10	-0.32	0.43	-0.54	1.03	0.83	0.19	0.97	0.16	0.61	1.58	0.12	0.99	-0.88	0.19	-0.73
Distancing	-0.15	0.55	2.85**	0.19	0.82	0.10	2.29*	0.01*	10.36***	2.68	0.01**	7.51***	-0.85	0.18	4.30***	1.44	0.19	4.45***	0.28	0.67	2.91**
Goal Oriented	-0.54	0.54	-0.77	0.29	0.89	-0.19	0.28	0.38*	0.43	-0.71	0.27	0.89	0.31	0.71	0.27	-0.98	0.29	-1.07	1.72	0.05*	1.89
Social Support	1.67	0.00**	1.38	1.09	0.17	1.34	4.21***	<0.001***	2.13*	0.14	0.28	-1.75	0.33	0.88	0.05	-2.07*	0.01**	-2.53*	1.81	0.00**	1.79
Avoidance Acceptance	0.61	0.23	-0.44	-0.92	0.61	-0.92	0.76	0.44	-2.57*	1.84	0.95	-1.69	2.45*	0.02*	1.06	1.51	0.16	0.87	0.63	0.44	-0.44

*** p≤0.001, ** p≤0.01, *p≤0.5. ADL: Activities of Daily Living; EWB: Emotional Well-Being; BD: Bodily Discomfort
III: control model, including all variables; IV: including all demographics except gender; V: including all demographics and the coping strategies, but without anxiety and depression
